# The sixth revolution in pediatric vaccinology: immunoengineering and delivery systems

**DOI:** 10.1038/s41390-020-01112-y

**Published:** 2020-09-14

**Authors:** Dheeraj Soni, Sharan Bobbala, Sophia Li, Evan A. Scott, David J. Dowling

**Affiliations:** 1 Precision Vaccines Program, Division of Infectious Diseases, Boston Children’s Hospital, Boston, MA USA; 2 Department of Pediatrics, Harvard Medical School, Boston, MA USA; 3 Department of Biomedical Engineering, Northwestern University, Evanston, IL USA

## Abstract

**Abstract:**

Infection is the predominant cause of mortality in early life, and immunization is the most promising biomedical intervention to reduce this burden. However, very young infants fail to respond optimally to most vaccines currently in use, especially neonates. In 2005, Stanley Plotkin proposed that new delivery systems would spur a new revolution in pediatric vaccinology, just as attenuation, inactivation, cell culture of viruses, genetic engineering, and adjuvantation had done in preceding decades. Recent advances in the field of immunoengineering, which is evolving alongside vaccinology, have begun to increasingly influence vaccine formulation design. Historically, the particulate nature of materials used in many vaccine formulations was empiric, often because of the need to stabilize antigens or reduce endotoxin levels. However, present vaccine delivery systems are rationally engineered to mimic the size, shape, and surface chemistry of pathogens, and are therefore often referred to as “pathogen-like particles”. More than a decade from his original assessment, we re-assess Plotkin’s prediction. In addition, we highlight how immunoengineering and advanced delivery systems may be uniquely capable of enhancing vaccine responses in vulnerable populations, such as infants.

**Impact:**

- Immunoengineering and advanced delivery systems are leading to new developments in pediatric vaccinology.
- Summarizes delivery systems currently in use and development, and prospects for the future.
- Broad overview of immunoengineering’s impact on vaccinology, catering to Pediatric Clinicians and Immunologists.

## Introduction

The impact of vaccination on the health of the world’s peoples is hard to exaggerate. With the exception of safe water, no other modality has had such a major effect on mortality reduction and population growth (Dr. Stanley A. Plotkin, MD, Vaccines, 1988).

So what is my choice for the sixth revolution?… I suggest that it will be New Delivery Systems (Dr. Stanley A. Plotkin, MD, Pediatric Academic Societies Meeting, May, 2004).

The goal of vaccination is to trigger an immune response that reduces the risk of infection and prevents disease.^1^ Initially, delivery systems were redundant since the majority of vaccines employed live attenuated organisms, which were often particulate in nature and inherently carried the abundant and necessary immune-stimulating signals. However, as vaccinology moved towards the development of defined antigens such as inactivated, subunit, and purified recombinant proteins and peptides, which are inadequate to trigger an immune response alone, the use of novel delivery systems became crucial. Historically, antigen stabilization (i.e., adsorption onto alum) guided vaccine formulation design. Therefore, the particulate nature of materials used in many early vaccine formulations was empiric. In this context, inclusion of alum adjuvants has been key to the acceptable efficacy of these subunit vaccine formulations, even though alum-adjuvanted vaccines usually require multiple doses for optimal protection.^2^ Second-generation efforts employed more characterized materials, such as the biodegradable synthetic polymer poly(d,l-lactic- co-glycolic acid) (PLGA), which is a widely investigated nanoparticle adjuvant for controlled and effective delivery of vaccine antigens, including synthetic peptides. These are typically produced as solid-core nanoparticles ranging from 50 to 500 nm in size, with antigens entrapped or adsorbed on the surface of the particles.^3^

More recently, advances in the field of immunoengineering, which are developing alongside vaccinology, have begun to greatly influence vaccine formulation design.^4,5^ Due to the wide differences in mechanisms of action of various non-adjuvanted and adjuvanted vaccines, vaccine formulation development has become a major consideration for vaccinologists and pharmaceutical companies.^6^ These considerations include (1) physicochemical characteristics of the formulation, (2) adjuvant chemical structure, (3) proposed route of administration, and (4) short- and long-term formulation stability.^5^ Specifically, vaccine delivery systems can now be engineered to mimic the size, shape, and surface chemistry of pathogens,^7^ which are often referred to as “pathogen-like particles”. Nanoparticle formulations can now be manufactured to target subsets of immune cells and specific subcellular compartments.^8–10^ One aspect consistent across current and future novel vaccines is the need to determine their ability to instruct adaptive immunity through the manipulation of antigen-presenting cells (APCs). As a part of their function as professional APCs, dendritic cells (DCs) can integrate information from extrinsic stimuli (e.g., components of pathogens or vaccines) and orchestrate these signals into appropriately regulated adaptive immune responses.^11^ As such, immunoengineering, adjuvant and antigen discovery, vaccine delivery, and increased knowledge of human immune responses are fueling a revolution in vaccinology.^12^

**Fig. 1:**
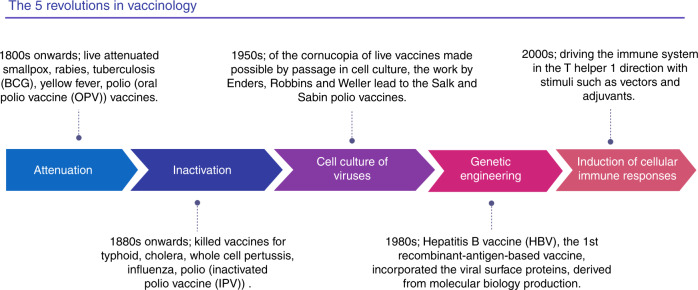
The five revolutions in vaccinology. *Attenuation*: 1800s onwards; live attenuated smallpox, rabies, tuberculosis (BCG), yellow fever, polio (oral polio vaccine (OPV)) vaccines. *Inactivation:* 1880s onwards; killed vaccines for typhoid, cholera, whole-cell pertussis, influenza, polio (inactivated polio vaccine (IPV)). *Cell culture of viruses*: 1950s; of the cornucopia of live vaccines made possible by passage in cell culture, the work by Enders, Robbins, and Weller lead to the Salk and Sabin polio vaccines. *Genetic engineering*: 1980s: Hepatitis B vaccine (HBV), the first recombinant-antigen-based vaccine, incorporated the viral surface proteins, derived from molecular biology production. *Methods to induce cellular immune responses*: 2000s; driving the immune system in the T helper 1 direction with stimuli such as vectors and adjuvants.

**Fig. 2:**
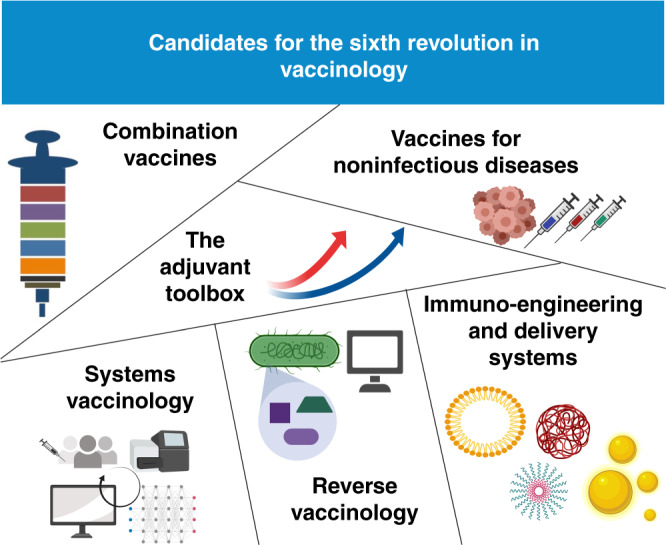
Candidates for the sixth revolution in vaccinology. *Combination vaccines*: simultaneous administration of vaccines to target multiple diseases. *The adjuvant toolbox*: ranging from small-molecule adjuvants to combination adjuvants. *Vaccines for non-infectious diseases*: new treatments for tumors, allergy, or non-infectious disorders (e.g., prevention of drug overdose). *Systems vaccinology*: Systems biology approaches to identify predictors of vaccine efficacy and explore new insights about protective immunity. *Reverse vaccinology*: Bioinformatics aided vaccine design from pathogenic genetics. *Immunoengineering and delivery systems*: Delivering precise materials for specific activation of immune system (right time, right place, right size, right shape, etc.).

**Fig. 3:**
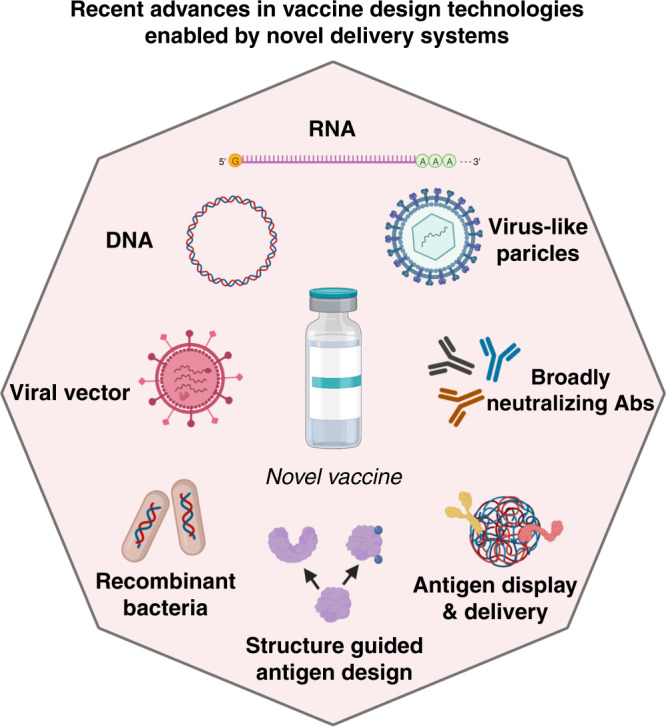
Recent advances in vaccine design technologies enabled by novel delivery systems. *DNA*: Plasmid contains DNA sequences encoding the pathogenic antigen(s). *RNA*: mRNA strand encodes for pathogenic antigen(s). *Virus-like particles*: multiprotein structures mimicking pathogenic virus however lacking their genome. *Broadly neutralizing Abs*: target conserved epitopes of the pathogen, regardless of mutation in pathogen (e.g. passive immunization with Palivizumab (RSV treatment)). *Antigen display and delivery*: antigen presentation on self-assembing nanoparticles to enhance humoral responses (e.g. multivalent display, co-display, immunomodulation, and genetic delivery). *Structure-guided antigen design*: structural manipulations of vaccine antigens (e.g. conformational stabilization, epitope focusing, epitope scaffolding, and antigenicity modification). *Recombinant bacteria*: recombinant bacterial vector/bacteria carries pieces of the pathogen. *Viral vector*: recombinant viral vector/another virus carries pieces of the pathogen.

More than 15 years ago, Stanley Plotkin^13^ identified the five crucial technical advances that revolutionized the field of vaccinology. These were attenuation (1800s; e.g., live attenuated rabies), inactivation (1880s onwards; e.g., killed vaccines for typhoid and cholera), cell culture of viruses (1950s; cultured poliovirus), genetic engineering (1980s; recombinant protein-based hepatitis B vaccine), and the induction of cell-mediated immunity (i.e., via adjuvantation) (Fig. 1). Dr. Plotkin identified six candidate areas that may start a possible sixth revolution (Fig. 2). These ranged from the combinatorial employment of older vaccine strategies to newer methods such as reverse vaccinology.^13^ Ultimately, the pioneering and burgeoning science driving the use of “Delivery Systems” was his choice to lead the way. Interestingly, there has also been remarkable progress in vaccine design and technologies including structure-guided antigen design, broadly neutralizing antibodies and promising novel vaccine platforms (DNA, mRNA) (Fig. 3). Many of these advances are reliant, at least in part, on optimized delivery.^14^ After nearly a decade and a half, in this review we reevaluate Dr. Plotkin’s vision to assess whether his prediction has been validated or is yet unanswered.

## Contemporary immunoengineering systems

Recent advances in the nascent field of immunoengineering may allow for the design of vaccine delivery systems that can potentially accelerate the development of novel and effective early life vaccination strategies.^15^ Immunoengineering may guide vaccine design^4,5^ by enabling the targeting of DCs,^16^ specifically via synthetic vaccine delivery systems that mimic the size, shape, and surface chemistry of pathogens.^7^ From an immunoengineering stance, the ideal vaccine formulation is (a) customizable, (b) based upon a scalable synthetic vaccine platform with (c) tunable release kinetics and can (d) stably encapsulate controllable amounts of molecules possessing diverse chemistry and water solubility. Such macro- and nanoscale- theranostic (i.e. therapeutic and diagnostic) materials would enable sustained and targeted delivery of complex antigen/adjuvant combinations via nanocarriers for hours, days, weeks, or months. By providing control over intracellular delivery of antigens and adjuvants to APCs, immunoengineering can more effectively activate innate immunity and moreover APCs to boost the adaptive immune response.^4^ Nanoparticle delivery systems can increase intracellular delivery to APCs by mimicking the morphology of viruses and by incorporating targeting ligands.^9,17^ In addition, the size, charge, and morphology of nanoparticles also affect lymph node trafficking.^18–20^ Control of nanoparticle delivery to intracellular compartments can be used to modulate the immune response post-vaccination. Delivery of antigens to the cytosolic or endocytic compartments respectively leads to antigen presentation via major histocompatibility complex (MHC) class I or MHC class II to prime distinct T cell subsets.^4^ This can be achieved by engineering antigen-loaded nanoparticles to induce endosomal or lysosomal escape, leading to antigen cross-presentation.^21,22^ Furthermore, the targeting of nanoparticles carrying Toll-like receptor (TLR) agonists to endosomes leads to activation of endosomal TLRs, promoting strong Th1-type responses.^23–25^ Activation of the inflammasome through cytosolic NOD-like receptors (NLRs) can lead to a self-adjuvant effect by the nanoparticle vehicle itself that promotes adaptive immunity.^26–28^ Lastly, nanoparticles containing vaccines can be used to control the duration of delivery through synchronous and sustained release of antigens and adjuvants.^29,30^ Delivery kinetics can be further modified using strategies such as incorporating nanoparticles into hydrogels or intradermal microneedle delivery systems.^29–31^ These sustained release systems can allow for the development of single-dose vaccines.^32^ Thus, pathogen-mimicking nanoparticles can be engineered to enhance the immune response by controlling when and where vaccine components are delivered intracellularly to APCs.^15^ A plethora of particulate delivery systems for immunoengineering have been developed, which are summarized further in this review.

## Lipid-based delivery systems

### Liposomes

Liposomes have been considered a dominant vaccine delivery platform because of their superior adjuvant properties^33^ and versatility in accommodating vaccine components.^34,35^ Liposomes are spherical vesicle structures comprising a phospholipid bilayer shell and aqueous lumen with sizes ranging from a few nanometers to several microns.^33,36^ These vesicles have been engineered to optimize their immunological role using several approaches including modulation of size and charge,^37,38^ surface decoration with immune cell recognition epitopes,^39,40^ and PEGylation to enhance in vivo circulation times.^41^ An adjuvanted-liposomal vaccine formulation AS01_B_ is marketed by GlaxoSmithKline as a component of Shingles vaccine (Shingrix)^42^ and is also in human clinical trials for malaria^43^ and HIV^44^ vaccines. Of note, cationic liposomes have shown high adjuvanticity,^45^ and a cationic liposomal formulation CFA01 is currently being tested in human clinical trials for HIV^46^ and tuberculosis vaccines.^47^

### Virosomes

Virosomes are nanosized phospholipid vesicles with membranes incorporating viral envelope proteins, typically produced from reconstituted empty envelopes of influenza viruses.^48^ They are utilized as both a vaccine carrier system and as an adjuvant, where the antigen of interest is either adsorbed or encapsulated within the lumen.^49,50^ Virosomes have been reported to induce both cellular and humoral immunity through efficient presentation of antigen via both MHC class I and II proteins;^48^ however, their exact mode of action is still unclear. Virosome-based influenza vaccines are licensed in Europe (as Inflexal) and as adjuvants for hepatitis A vaccine (as Epaxal).

### ISCOMs

Immune-stimulating complexes (ISCOMs) are spherical cage-like nanoparticles (~40 nm) formed via self-assembly of a mixture of the saponin adjuvant Quil A, cholesterol, phospholipids, and antigens.^51^ ISCOMs in the absence of an antigen are called ISCOMATRIX and can be mixed with any antigen of interest.^52^ ISCOMs have been reported to stimulate enhanced cellular responses with lower antigen doses through enhanced antigen cross-presentation.^53^ However, the role of individual components in generating these immune responses is unknown. At present, ISCOM technology has been approved only for veterinary vaccines;^54^ however human clinical trials are currently ongoing for the development of melanoma vaccines.^55^

## Polymer-based delivery systems

Modern-day vaccine research is highly dependent on flexible engineering strategies such as tunable immune cell recognition epitopes,^56^ morphological diversity,^9^ and precise intracellular delivery.^8^ Polymer-based systems have been considered front-runners for incorporating these strategies as compared to their lipid-based counterparts. Polymeric delivery vehicles are mainly classified as solid-core or self-assembled. These delivery vehicles have an additional advantage of greater physicochemical and in vivo stability as compared to lipid-based systems.^36^

### Solid-core particles

PLGA micro- and nanoparticles have been widely explored for vaccine delivery applications because of their ease of fabrication, amenability to surface modification, encapsulation efficiency of both hydrophilic and hydrophobic vaccine components, and modifiable release properties.^57,58^ Several recent studies suggest that the depot forming ability of PLGA microspheres at the injection site could be a potential single-shot vaccination strategy.^59,60^ The ability of PLGA particulate vaccines to generate strong cytotoxic responses make them great candidates for the development of vaccines against infectious diseases^61,62^ and cancer.^63^

Another interesting solid-core vaccine delivery vehicle, pluronic-stabilized polypropylene sulfide (PPS) nanoparticles, have been developed.^64,65^ These particles contain a hydrophobic PPS core, which becomes hydrophilic under oxidative conditions of cell endosomes, promoting intracellular release. Reddy et al.^65^ showed highly efficient transportation of the PPS nanoparticles (25 nm) through lymphatic capillaries to target half of the lymph node resident DCs and unveiled the role of PPS surface chemistry in activating the complement cascade. Further, PPS nanoparticles conjugated with a model protein antigen OVA stimulated both cellular and humoral responses.^65,66^

### Self-assembled particles

The self-assembly of polymeric structures is often achieved using block copolymers, which comprise linked hydrophobic and hydrophilic polymer blocks.^67^ The application of diverse block copolymer chemistries and self-assembled morphologies has been evaluated for immunomodulation.^68,69^ The simplest morphology that can be attained is a micelle, which contains a hydrophobic core and a hydrophilic corona.^70^ Polylactide (PLA)-based block copolymer micelles have been widely explored for vaccine delivery. Specifically, polyethylene glycol (PEG)-*b*-PLA block copolymer micelles have been reported to show excellent biocompatibility, immunomodulatory ability, and success in inducing cellular immunity.^71^ Recently, environment-responsive micelles consisting of pH-^72^ or oxidation-responsive^73^ block copolymers have shown great promise in releasing vaccine components inside cell lysosomes. There is also great interest in developing polymersomes, which are self-assembled polymeric vesicles analogous to liposomes, for delivering vaccines and inducing cellular immunity. PEG-*b*-PPS, an oxidation-responsive block copolymer, has been reported to self-assemble into monodisperse polymersomes and encapsulate a wide range of antigens and adjuvants.^25,68^ Stano et al.^66^ reported that PEG-*b*-PPS polymersomes loaded with antigen (OVA) and adjuvant (CpG) induced enhanced CD4^+^ T cell responses in the spleen and lymph nodes. Furthermore, PEG-*b*-PPS block copolymers have been engineered to self-assemble into other diverse morphological structures. These block copolymers with PEG weight fractions (*f*_PEG_) of 0.1, 0.25, 0.38, and 0.45 self-assemble to form bicontinuous nanospheres (polymeric cubosomes),^74^ vesicles (polymersomes),^75^ cylindrical micelles (filomicelles),^31^ and micelles,^10^ respectively. Recent studies revealed that these structures have differential organ biodistributions and immune cell uptake in vivo,^9,76^ which makes them strong contenders for the development of new rationally designed immunotherapies and vaccines.

## Inorganic nanoparticles

Aluminum salts (Alum) including aluminum hydroxide and aluminum phosphate are the most commonly used adjuvants in human vaccines.^77^ In aluminum salt-based vaccine formulations, antigens are adsorbed to the highly charged aluminum hydroxide or aluminum phosphate gel.^78^

The molecular mechanisms by which Alum interacts with the human immune system continues to be studied and involves multiple pathways, both direct and indirect. The majority of studies suggest that the adjuvant action of aluminum salts is mediated by activation of the NLRP3 inflammasome.^79^ Alum may directly or indirectly trigger innate immunity via activation of inflammasome complexes, required for the processing of IL-1 family pro-inflammatory cytokines. This process is most likely NLR-mediated, since the adjuvant effects of Alum are not impaired in the absence of key TLR-dependent signal transduction adaptor molecules MyD88 and TRIF in knockout mice. Secondly, Alum enhances delivery of antigen to APCs as particulate vaccine formulations more readily interact with DCs and macrophages than soluble formulations of antigens alone.^7^ Crystalline Alum binds lipids on the surface of DCs and triggers a cellular activation cascade leading to initiation of an immune response, but without itself being internalized by the cells.^80^ Thirdly, Alum-induced cell death seems to modulate the local milieu in favor of enhanced adaptive immune stimulation. The release of damage-associated molecular patterns, such as uric acid and dsDNA, act as autologously derived auto-adjuvants.^81^

Induction of humoral immunity is a hallmark feature of aluminum-containing adjuvants. For instance, Alum-adjuvanted vaccines often require multiple doses for induced protection,^2^ and drive Th2- over Th1-polarized immunity. Presently, there are multiple licensed pediatric vaccines, such as diphtheria, tetanus, and hepatitis vaccines, listing Alum as essential to produce effective antibody (Ab) titers. Several new strategies have been considered to modify these Alum particles for induction of cellular responses. For example, Liu et al.^82^ encapsulated alum colloid inside a yeast-derived β-glucan particle, which induced greater cellular immune responses. Another group, Wang et al.,^83^ made phospholipid bilayer-coated aluminum nanoparticles that were readily taken up by the APCs and stimulated antigen-specific cellular and humoral responses in vivo. However, there are also vaccinal antigens for which addition of Alum may not be necessary for effective immunogenicity. For example, Alum was excluded from the recent vaccine Menveo (MenACWY) due to failure of Alum to enhance improvement in serum bactericidal Ab titers during infant clinical trials.^84^

Gold nanoparticles are versatile systems that can be easily synthesized into different morphologies and are able to accommodate a diverse range of antigens and adjuvants onto their surface.^85^ In several studies, gold particles have been reported to have efficient accumulation in DCs and B cells, playing a critical role in generating cellular and humoral responses.^86,87^ Recent findings on gold nanoparticle-based cancer vaccines demonstrated that the photothermal ablation ability of gold particles alongside cellular cytotoxic responses can have a synergistic impact in cancer treatment.^88^ Additionally, inorganic particles like carbon nanotubes,^89^ mesoporous silica,^90^ and iron oxide nanoparticles^91^ have been widely explored as vaccine delivery vehicles. However, more understanding of their toxicity and safety profiles may be required for the advancement of these nanoparticles.

## Emulsions

Diverse water-in-oil emulsions, of which incomplete Freund’s adjuvant is the best-known, were originally evaluated in human trials during the mid-twentieth century.^92^ Emulsions are biphasic systems consisting of a water and an oil phase, where the interface is stabilized using a surfactant. Emulsions can be classified as simple emulsions (oil-in-water (O/W) and water-in-oil (W/O) type) or multiple emulsions (oil-in-water-in-oil (O/W/O) and water-in-oil-in-water (W/O/W) type).^93^ W/O emulsions were soon withdrawn from commercial development due to unacceptable reactogenicity (increased risk of cysts at the injection site) and lack of formulation reproducibility. This led to the development of O/W emulsions, in which oil droplets are present in a continuous aqueous phase. These systems are often utilized as vaccine adjuvants and antigens are adsorbed onto the emulsion droplets. Different mechanisms for the adjuvanticity of emulsions have been proposed including (1) slow release of antigen from a depot formed at the site of injection and (2) antigen uptake by recruited immune cells at the injection site.^94^ Generally, O/W emulsions create an “immune-competent environment” within the muscle after intramuscular injection, the mechanism of action of which is TLR-independent.^92^ For example, the adjuvant MF59 does not directly mature APCs, but rather induces the production of chemokines and immune modulatory proteins from monocytes, macrophages, granulocytes, and muscle cells that indirectly leads to increased migration of APCs to/from the site of injection and into draining lymph nodes. This cell recruitment is greater than that induced by Alum. MF59 may also instruct peripheral site monocyte differentiation into DCs and possibly induce the release of endogenous TLR agonists. Therefore, one major advantage of O/W adjuvants is the antigen dose-sparing potential.^95^ For the last 20 years, O/W emulsions have been popular adjuvants to enhance Ab responses in many seasonal and pandemic influenza vaccines licensed with European regulatory authorities (EMA),^95^ using MF59 (Fluid, Focetria), AS03 (Pandemrix), and AF03 (Humenza) as adjuvants.^92^ Currently, squalene oil-based O/W emulsions, MF59 and AS03, have been approved for human influenza vaccines.^96^

## Advanced delivery systems may be uniquely capable of enhancing vaccine responses in the very young

The majority of vaccination programs have historically focused on the pediatric age group. However, newborns display distinct immune responses, leaving them vulnerable to infections and impaired immunization owing to slow initiation, decreased magnitude of immunogenicity, reduced persistence of functional antibodies, and weak or absent cell-mediated responses.^2^ Early life vaccines thus often require multiple vaccine doses to be effective.^97^ Since vaccine development pipelines rarely tailor formulations (adjuvants, delivery systems, etc.) rationally for use in early life, it is critical to understand the optimization of vaccine efficacy by taking into account early life immune ontogeny.^98–100^ For example, early life vaccination against intracellular pathogens has proven difficult.^101^ Due to functionally distinct and delayed T cell-mediated immunity, newborns and young infants are highly susceptible to infection with intracellular pathogens, including bacteria such as *Listeria* spp. and *Salmonella* spp., viruses such as herpes simplex virus and respiratory syncytial virus,^102^ and intracellular pathogens of global significance such as HIV, tuberculosis, and malaria. Here, nanoparticle-based formulations may hold great promise for pediatric vaccine development.

Targeting the key deficiencies of newborn DCs relative to adult DCs may enable development of age-specific vaccine formulations to overcome sub-optimal immunization responses. Recently, we have combined such engineering and rational vaccine design approaches to develop a nanoparticle-based adjuvant and antigen-delivery system designed to be active in human newborns and infants. The design mimicked, and in some instances, exceeded the immunostimulatory effect of live attenuated vaccines.^68^ We employed PEG-*b*-PPS polymersomes, which are significantly more stable than liposomes, as the effective adjuvant- and antigen-delivery system. These polymersomes can be effectively engineered for bioresponsive intracellular payload delivery,^5^ making them highly advantageous for the specific targeting of endosomal receptors. This is notable, since the activation of endosomal pattern recognition receptors (PRRs), as compared to surface PRRs (e.g. TLR2 and TLR4), instructs more adult-like innate immune responses in newborn DCs. Small-molecule TLR8 agonists (e.g. imidazoquinoline) robustly activate newborn DCs but can result in systemic responses when delivered in soluble form. To overcome this and minimize off-target effects, we developed TLR8-agonist-encapsulating polymersomes, which demonstrated a preference for uptake by DCs relative to other cell populations following subcutaneous administration. Furthermore, such formulations hold substantial potential for early life immunization by serving as a dual antigen/adjuvant delivery system that mimics the enhanced neonatal innate and adaptive immune responses elicited by the live Bacille Calmette-Guerin (BCG) vaccine. Strikingly, when co-loaded with the *Mycobacterium tuberculosis* antigen 85B peptide 25, the TLR8-agonist containing polymersomes were comparable to BCG in inducing antigen-specific immune responses in human TLR8-expressing neonatal mice in vivo.^68^ This is promising, since BCG reduces the risk of disseminated early life tuberculosis by safely eliciting Th1-type neonatal immune responses and requires only a single dose at or shortly after the time of birth.

Another promising approach is the use of microstructures designed for controlled release of vaccine formulations in vivo. McHugh et al. recently developed a microstructure called StampEd Assembly of polymer Layers (SEAL). SEALs are small (≤200–400 μm) PLGA-based polymeric microdevices with complex geometries, which can be filled with soluble drug solutions and then completely sealed.^103^ Such structures can be rationally designed with additive manufacturing processes (i.e., three-dimensional (3D) printing). Importantly, depending on the formulation and chemical composition, these materials can be tuned to achieve continuous or delayed/pulsatile release kinetics.^104^ This was achieved by tuning the degradation of the materials (i.e., the copolymer ratio). As opposed to solid-particulate delivery systems formulated to deliver antigen continuously, which demonstrate an initial burst and then slow release thereafter, such “pulsatile vaccines” more closely resemble traditional vaccine schedules, which are based around the concept of priming followed by multi-booster injections, but with the advantage of requiring only a single immunization. This may be especially promising for combinatorial vaccination approaches that require multiple boosters, as commonly used in the pediatric setting,^105^ or induction of protective immunity that may rely on persistent pathogenic antigen exposure.^106^ These and other such technologies may ultimately open the way for single-injection vaccine strategies.^68,104^

## Reassessing vaccination schedules and expanding vaccine target populations

Most vaccination schedules throughout the world are designed for the pediatric age group. Notably, most schedules recommend children to be immunized against hepatitis B virus (and often tuberculosis through the BCG vaccine) starting in the neonatal period. Immunization to rotavirus, diphtheria, tetanus, pertussis, *H. influenzae* type b, *Streptococcus pneumoniae*, poliovirus, influenza, measles, mumps, rubella, varicella, hepatitis A virus, meningococcal, and human papillomavirus extend from 2 months of age up to adolescence.^105^ As the twenty-first century progresses, the current immunization schedule should remain flexible to incorporate newly invented vaccines as they become available with emerging and reemerging infectious diseases, such as meningococcus, influenza virus, group A streptococcus, *Helicobacter pylori*, and respiratory syncytial virus (possibly the most important infant vaccine still missing from this clinical schedule).

In addition, expanding the current schedule to cover more vaccine-preventable infectious diseases and including vaccine formulations incorporating novel immunoengineered delivery systems may be key tools to allow for an accelerated schedule approach (Table 1). As outlined above, with the exception of the hepatitis B virus vaccine and BCG (which in some countries are given at birth), present immunization schedules start mostly after 2 months of age.^105^ Consequently, the present immunization schedules do not induce protection against the majority of these diseases until the fifth–sixth months of life or later. This creates a period of vulnerability during the first 6 months of life, which is associated with significant mortality and morbidity. Therefore, current aspirational efforts often focus on the development of (a) single-dose immunization strategies for newborns that instruct lifelong protection to subsequent challenges or (b) optimizing maternal immunization as means to cover this susceptibility gap. Heterologous prime-boost vaccination strategies (e.g. an attenuated vaccine followed by an inactivated vaccine targeting the same pathogen) may also be advantageous in early life, especially if they incorporate the same antigen but distinct delivery systems. Current prime-boost regimens incorporating viral vectors or DNA vaccines, followed by a boost with a protein-based vaccine may be able to overcome distinct immune ontogeny in early life while also taking advantage of the relatively lower rates of reactogenicity in the neonatal and early infant periods. Furthermore, increased appreciation of immune ontogeny may inform development of rationally designed age-specific vaccine formulations. Future studies need to focus not only on safety and efficacy, but also on potential vaccine–vaccine interactions that can lead to interference, and possibly include adjuvants that more effectively enhance immune responses in early life. Innate immune memory, i.e. an alteration of reactivity in innate immune cells previously exposed to diverse stimuli, may also confer heterologous immunity that could be leveraged by some adjuvanted vaccine formulations in the future.^107^

**Table 1:** Potential uses and benefits of “novel delivery systems” to future early life vaccination strategies.

**New adjuvants**
• Instruct an accelerated, targeted, potent, and durable immune response in humans against pathogens (e.g. overcome pathogen diversity and immune evasion)
• Allow for dose sparing and reduced vaccine manufacturing costs, thereby increasing the global access to pediatric vaccines
• Dramatically increase the number of antigens per formulation/immunization
• Modulation of antigen delivery and persistence (i.e., single bolus vs. slow release formulations)
• Act as immunomodulators to enhance Th (e.g., T helper 1 [Th1] cell versus Th2) or achieve qualitative alteration of the immune response (CD8+ versus CD4+ T cells)
• Allow cell-mediated T cell vaccination strategies
• Capture synergy by using different adjuvant combinations
• Allow dendritic cell (and DC subset) targeting
**Reassessing the schedule**.
• Reduce number of immunizations (possibly to even a single dose)
• Reduce reactogenicity and improve safety with age-optimized formulations
• Locally and specifically circumvent suppressive innate immune ontogeny
• Enhance potential of therapeutic vaccines for early life allergies and chronic infectious diseases
• Modulate maternal immune system effects and/or microbiome on infant vaccine responses early in life
**Vaccine target populations**
• Expand the efficacy of licensed vaccines to neonates and preterm infants
• Develop dedicated vaccines for pregnant women
• Broaden administration route options, such as neonatal mucosal vaccination
• Broader vaccination for immunocompromised populations
• Reduce human reservoirs of infectious diseases, by improving vaccines designed for the elderly
• Polarize maternal responses to minimize interference with infant response to subsequent vaccination or infection
• Induction of un-natural immunity (i.e., broadly protective universal vaccines)
• Instruct heterologous immunity, thereby reducing overall mortality rates

Vaccination holds promise for primary prevention not only for neonates and infants but also for diverse age- and target groups, including adults, elderly, adolescents, pregnant women, preterm infants, fetuses, and people suffering from chronic and immune-compromising diseases. These groups represent heterogeneous populations, separated by genetic background, gender, pregnancy, microbiome, diet, lifestyle, poverty, risk of infectious diseases, and geography (those in the developed and developing world where future vaccines would be deployed) among others. Therefore, considering these broad differences, it is critical to understand how novel delivery systems may be utilized effectively to optimize vaccine efficacy.^12^

Two of foremost the target populations are pregnant women and preterm infants. Notably, immunity of preterm infants is distinct from newborns,^108^ rendering them particularly susceptible to infection.^109,110^ Every year, ~15 million newborns are born preterm worldwide, which is ~11% of all live births.^111^ Preterm infants demonstrate impairments in innate and acquired immunity, significantly less maternal-derived Ab than term infants, and a higher risk of infection-induced disability and death.^109^ Early life immunization to close the window of disease vulnerability may be key to preventing these infections, but immune responses to subunit vaccines are impaired among preterm infants.^105^ For example, although administered at birth to term infants, Hepatitis B virus immunization is often delayed in preterms due to reduced immunogenicity in this population.^112^ Similarly, preterm infants demonstrate impaired serotype-specific immune responses to pneumococcal conjugate vaccine (PCV)-7.^113^ Novel use of preexisting and clinical licensed vaccine formulations may shed light on areas of optimization. Co-administration of BCG with HBV, for example, significantly enhanced anti-HBV IgG titers in mouse models of both term and preterm birth, equally, but not in adult mice.^108^ Accordingly, there is an unmet need for safe and immunogenic vaccines for preterm infants, including those targeting HBV and pneumococcus.

Maternal vaccination has emerged as a favorable public health approach in the past decade. This approach is highly promising as it may decrease maternal, fetal, and neonatal susceptibility to infections.^114^ While implementation may vary by region, currently a number of vaccines are universally recommended/indicated during pregnancy. These include vaccines against tetanus, influenza, and pertussis, along with new vaccines for group B streptococcus and respiratory syncytial virus that are currently being developed to prevent neonatal infections.^115^ As the efficacy of maternal vaccines significantly relies on active transport of antibodies at the maternal–fetal interface (through the placenta using an Fc receptor and concentrated in the fetus), antigen/adjuvant delivery systems may become key tools in expanding these efforts to cover a broader range of diseases. In addition, adjuvanted vaccine formulations may be specifically designed to polarize maternal responses to minimize interference with infant responses to subsequent vaccination or infection. Lastly, passive immunization (via the use of monoclonal Abs) may also be considered a relevant tool in areas where no vaccine currently exists. This includes the mAb Palivizumab (brand name Synagis), produced by recombinant DNA technology for the prevention of RSV infections, especially in at-risk preterm infants.^116^

## Conclusions and future directions

Modern vaccine design and development strategies endeavor to integrate knowledge of formulation composition and understanding of immunostimulatory mechanisms to generate immune responses otherwise unachievable in relevant target populations.^98,117^ Rational vaccine design approaches, employing novel immunoengineering strategies, and targeted delivery systems may allow for the controlled preparation of vaccine formulations of the desired immunostimulatory properties, particulate size, and antigen load, all of which can greatly improve safety and limit systemic toxicities due to their targeted nature.^118^ These potential advances may be key to tailored vaccines capable of matching unique characteristics of the developing immune system during the neonatal period and infancy.^2^ Plotkin^13^ highlighted various candidates for the sixth revolution of vaccinology, including the expanded use of combination vaccine strategies, new adjuvants, vaccines for non-infectious diseases, and the advent of systems vaccinology. Ultimately, he settled on new delivery systems. Interestingly, just as the breakthrough in cell culture expanded the role of attenuation and killed vaccine strategies, breakthroughs in immunoengineering and novel delivery systems have led to multitudes of promising vaccine platforms with great potential to advance innovation in pediatric vaccinology.
